# Severe influenza cases in paediatric intensive care units in Germany during the pre-pandemic seasons 2005 to 2008

**DOI:** 10.1186/1471-2334-11-233

**Published:** 2011-08-31

**Authors:** Andrea Streng, Veit Grote, Johannes G Liese

**Affiliations:** 1 Department of Paediatric Infectious Diseases and Immunology, University Children's Hospital, Julius-Maximilians-University, Würzburg, Germany; 2 Department of Immunology and Infectiology, Dr. von Haunersches Kinderspital, University Children's Hospital, Ludwig-Maximilians-University, Munich, Germany

## Abstract

**Background:**

Data on complications in children with seasonal influenza virus infection are limited. We initiated a nation-wide three-year surveillance of children who were admitted to a paediatric intensive care unit (PICU) with severe seasonal influenza.

**Methods:**

From October 2005 to July 2008, active surveillance was performed using an established reporting system for rare diseases (ESPED) including all paediatric hospitals in Germany. Cases to be reported were hospitalized children < 17 years of age with laboratory-confirmed influenza treated in a PICU or dying in hospital.

**Results:**

Twenty severe influenza-associated cases were reported from 14 PICUs during three pre-pandemic influenza seasons (2005-2008). The median age of the patients (12 males/8 females) was 7.5 years (range 0.1-15 years). None had received vaccination against influenza. In 14 (70%) patients, the infection had been caused by influenza A and in five (25%) by influenza B; in one child (5%) the influenza type was not reported. Patients spent a median of 19 (IQR 12-38) days in the hospital and a median of 11 days (IQR 6-18 days) in the PICU; 10 (50%) needed mechanical ventilation. Most frequent diagnoses were influenza-associated pneumonia (60%), bronchitis/bronchiolitis (30%), encephalitis/encephalopathy (25%), secondary bacterial pneumonia (25%), and ARDS (25%). Eleven (55%) children had chronic underlying medical conditions, including 8 (40%) with chronic pulmonary diseases. Two influenza A- associated deaths were reported: *i) *an 8-year old boy with pneumococcal encephalopathy following influenza infection died from cerebral edema, *ii) *a 14-year-old boy with asthma bronchiale, cardiac malformation and Addison's disease died from cardiac and respiratory failure. For nine (45%) patients, possibly permanent sequelae were reported (3 neurological, 3 pulmonary, 3 other sequelae).

**Conclusions:**

Influenza-associated pneumonia and secondary bacterial infections are relevant complications of seasonal influenza in Germany. The incidence of severe influenza cases in PICUs was relatively low. This may be either due to the weak to moderate seasonal influenza activity during the years 2005 to 2008 or due to under-diagnosis of influenza by physicians. Fifty% of the observed severe cases might have been prevented by following the recommendations for vaccination of risk groups in Germany.

## Background

Influenza belongs to the most frequent vaccine-preventable diseases. Clinical manifestations of influenza infections range from illness with asymptomatic, atypical (i.e., gastro-intestinal) or oligosymptomatic disease to severe toxic progression resulting in death [1,2]. Elderly people and infants account for the highest morbidity [1,3,4].

Complications of influenza may occur at any age, affecting most severely patients with chronic underlying diseases, as chronic pulmonary diseases, diseases of the circulatory system, metabolic diseases, or immunodeficiency. The most severe complication is peracute fatality in adolescents and young adults caused by acute circulatory failure due to myocarditis or fulminant necrotising influenza-associated pneumonia. A frequently reported complication is a secondary bacterial pneumonia. Other known complications include encephalitis, otitis, and neuromuscular diseases [1,3,4].

Surveillance studies from several industrialized countries reported an incidence of paediatric hospitalisations associated with seasonal influenza of 11-154/100,000 children < 5 years of age, and an incidence of 1-13/100,000 children treated for influenza in paediatric intensive care units (PICUs) [5-7]. For the influenza season 2003-2004, 153 influenza-associated deaths among children were reported in the United States; 47% of those occurred in children without underlying chronic disease [8].

In Germany, only limited epidemiological data on hospitalisations and complications of seasonal influenza in children is available. In the population-based PRIDE study, conducted in 11 paediatric practices and 4 hospitals on children < 3 years of age from November 1999 to October 2001, annual incidence of influenza-associated hospitalisations was estimated as 120/100,000 [9]. In another population-based study conducted 1996-2001 in two paediatric hospitals, incidence was calculated as 123/100,000 children < 6 years of age, and 53/100,000 in children < 17 years of age [10]. However, both studies were too small to detect rare severe influenza-associated complications and fatalities resulting in PICU admittance.

The objective of this study was the nation-wide detection of severe influenza virus infections and fatalities in children, and to identify possible risk factors for complications. These data are important in the current discussion of a possible extension of yearly influenza vaccination from a risk-based strategy to general vaccination of all children. The study was conducted before the occurrence of the new influenza A (H1N1) 2009 pandemic in Germany.

## Methods

### Study population

The study population included all children and adolescents < 17 years of age in Germany, with on average 13,166,000 children and adolescents during the years 2005-2008, including on average 3,497,000 children < 5 years of age (German Federal Statistical Office, Germany, 2006-2009).

### Reporting system

Prospective, active surveillance to capture severe influenza-associated complications and fatalities was performed from October 2005 to May 2008, covering three influenza seasons. Patients fulfilling the study case definition were included through the Surveillance Unit for Rare Paediatric Diseases in Germany (ESPED), a nation-wide voluntary reporting system covering 448 paediatric hospitals and departments. These hospitals contain approximately 21,500 beds, including about 2,650 paediatric intensive care beds (National Hospital Statistics, 2008). ESPED actively collected monthly reports on the occurrence of severe influenza-associated cases requiring intensive care treatment from all hospitals. Monthly reporting was requested even if no cases were identified. For identified cases, the hospitals were asked to return anonymous, structured questionnaires on basic demographic characteristics, underlying chronic medical conditions, influenza vaccination status, and influenza diagnostics, symptoms, treatment, pre-defined complications, and sequelae. The reporting physician filled in the questionnaire according to the information from the medical chart. If feasible, hospitals provided additionally anonymous medical discharge letters or autopsy results.

### Case definition

All children and adolescents < 17 years of age fulfilling the following criteria for severe paediatric influenza-associated infections were included: *i) *laboratory-confirmed influenza (antigen test or PCR or virus isolate) within one week before or after start of influenza symptoms, and *ii) *hospitalisation to a PICU with need for intratracheal/CPAP ventilation and/or one of the following diagnoses: encephalitis/encephalopathy, bronchitis/bronchiolitis, complicated febrile seizure, acute respiratory distress syndrome (ARDS), influenza-associated pneumonia, secondary bacterial pneumonia, status asthmaticus, sepsis, myocarditis, apnoea/bradycardia, or *iii) *influenza-associated death.

### Data analysis

All data were entered into a Microsoft Access 2000 database and, after plausibility checks, transferred into SPSS 15.0 for statistical analysis. Data were analysed descriptively (percentages or median with inter-quartile range, IQR; reported percentages refer to the number of available data per variable). Comparisons among groups were assessed for significance (p < 0.05, two-sided) using Pearson's Chi^2^-test or, where appropriate, Fisher's Exact test for categorical data. Continuous data were assessed using the Mann-Whitney U-test. Due to the small number of cases per subgroup, the evidence of the statistical comparisons was limited.

### Ethical considerations

The study was reviewed and approved by the Bavarian Data Protection Office, Munich, Germany. The institution agreed that patient informed consent was not needed for this surveillance study.

## Results

### Participating study sites and recruitment

Twenty (5%) hospitals reported 29 patients. The reporting hospitals were situated in 9 (56%) of the 16 federal states of Germany. Of the 29 reports, 9 (31%) had to be excluded from the analysis: for 3 patients, the hospitals did not provide information on the inclusion criteria, 3 patients had no laboratory-confirmed influenza diagnosis, 1 patient was older than 16 years of age, and 2 patients had been reported twice.

### Patient characteristics and duration of hospitalisation

A total of 20 valid patients reported from 14 (4%) hospitals were identified, 9 (45%) in the season 2005/2006, 6 (30%) in 2006/2007, and 5 (25%) in 2007/2008. Seasonal distribution peaked in March 2006, and in February 2007 and 2008. Twelve (60%) of the 20 children were male. The median age was 7.5 years (IQR 0.5-12.75, range < 1 month to 15 years of age); 9 (45%) children were < 5 years of age, including 5 (25%) children < 1 year of age.

Duration of hospital stay varied from < 1 to 96 days (median 19, IQR 12-38), duration of PICU stay was between < 1 and 50 days (median 11, IQR 6-18). In 70% of patients, PICU admission occurred at the day of hospital admission or within one day afterwards (range 0-10 days). In one child, the influenza infection was assumed to be nosocomial, with start of influenza symptoms and transfer to the PICU 9 days after initial hospitalisation due to complicated pneumonia caused by *Mycoplasma pneumonia*e.

### Virological and microbiological diagnostics

There were 14 (70%) cases of seasonal influenza A and 5 (25%) cases of influenza B; in one (5%) patient influenza infection was confirmed but the influenza type was not available. Influenza subtypes were determined for 3 samples, with seasonal influenza A (H1N1) in two patients and influenza A (H3N2) in one child. Influenza virus infection was determined by antigen testing in 8 (40%), PCR in 4 (20%), both antigen testing and PCR in 7 (35%) patients, and PCR combined with virus isolation in one (5%) patient. Most frequently, tracheal (30%) or pharyngeal (20%) secretions, nasopharyngeal swabs (15%) or a combination of these materials (20%) were used for diagnosis. In 2 (10%) children influenza infection was confirmed by serum diagnostics and in one (5%) patient from lung biopsy material after fatal outcome.

CRP values were available from 18 children (median 8.4 mg/dl, IQR 3.9-23.1). Laboratory-confirmed co-infections were reported in a total of 13 (65%) children (4 viral, 7 bacterial, 2 fungal; Table 1). Diagnostic analysis for simultaneous respiratory syncytial virus (RSV) infection was performed in 10 (50%) children; in 4 with positive results. All 4 children diagnosed with both influenza and RSV suffered from pneumonia and/or bronchitis/bronchiolitis and needed PICU treatment for a median of 9 days (IQR 4-13); three were < 1 year of age. Microbiological diagnostics were performed from blood cultures of 17 (85%) patients (3 positive with *Staphylococcus sp*.), from cerebrospinal fluid in 3 (15%) children (one positive post-mortem with *Streptococcus pneumoniae*), and from pleural fluid in 2 (10%) children (1 positive with *Staphylococcus aureus*; Table 1). Other positive results were mainly reported from tracheal aspirates in patients receiving intratracheal ventilation (Table 1). Of seven patients with bacterial co-infections, three were infected with two different bacterial species. Patients with co-infection by bacteria or fungi were treated at the PICU for a median of 18 days (IQR 12-30), and, thus, twice as long compared to patients without such co-infections (median 9 days, IQR 5-11; p = 0.01).

**Table 1:** Co-infections (viral, bacterial, fungal; culture-confirmed) in children with severe seasonal influenza - Germany, 2005-2008 *

Co-infection	Infectious agent	Diagnostics/material	No. of children (%)
***Viral***	Respiratory Syncytial Virus (RSV)	RSV-test (antigen, PCR, or immunofluorescence)	4 (20)
***Bacterial - gram positive***	*Staphylococcus aureus*	Pleural fluid (1), tracheal aspirate (2)	3 (15)
	*Staphylococcus epidermis*	Blood culture	2 (10)
	*Staphylococcus hominis*	Blood culture	1 (5)
	*Streptococcus pneumoniae*	Liquor and brain tissue (post-mortem)	1 (5)
	*Enterococcus sp.*	Central venous catheter/blood culture	1 (5)
***Bacterial - gram negative***	*Hafnia alvei*	Tracheal aspirate	1 (5)
***Bacterial - mycoplasma-like***	*Mycoplasma pneumoniae.*	Serology	1 (5)
***Fungal***	*Candida albicans*	Tube tip	1 (5)
	*Aspergillus sp.*	Tracheal aspirate	1 (5)

* Data on co-infections reported in 13 (65%) of 20 children; percentages refer to the number of all 20 patients. Multiple nominations were possible. *sp*: species; not further specified

### Clinical symptoms, radiological diagnoses and treatment

A total of 158 clinical signs and symptoms at PICU admittance were reported from 18 patients (71 (45%) respiratory, 38 (24%) gastro-intestinal, 49 (31%) neurological; Table 2). The most frequently reported symptoms were tachypnoea (16), feeding difficulties (15), and fever (15).

**Table 2:** Clinical signs and symptoms of severe seasonal influenza in children at PICU admittance - Germany, 2005-2008 *

Clinical signs and symptoms	No. of children (%)
Tachypnoea	16 (89)
Cough	14 (78)
Rhinitis	14 (78)
Conjunctivitis	14 (78)
Apnoea	13 (72)
Refusal of food or drink	15 (83)
Diarrhea	12 (67)
Vomiting	11 (61)
Fever ≥ 38°C	15 (83)
Clouding of consciousness	14 (78)
Headache	10 (56)
Pain in the limbs	10 (56)

* Data on symptoms were available from 18 children; percentages refer to the number of the available patients. Multiple nominations were possible.

A chest radiograph was performed in 17 (85%) and was abnormal in 16 (80%) patients. Reported radiological diagnoses were pneumonia (8), ARDS (2), pneumothorax (2), pleural effusion (2), mediastinal emphysema (1), pericardial effusion (1), and severe pulmonary haemorrhage (1).

Oseltamivir was administered to 10 (50%) children for a median duration of 5 days (IQR 4.5-5.25); treatment started in 5 children at the day of PICU admittance, in one child one day and in 4 children 5-12 days after PICU admittance. The delay between start of symptoms and oseltamivir treatment was between 1 and 15 days. A total of 18 (90%) children were treated intravenously with antibiotics, for a median of 13 days (IQR 7-15). Catecholamines were administered in 8 (40%) children for a median of 4 days (IQR 2-14). Inhalation with bronchodilators was required by 12 (60%) children. A total of 10 (50%) children needed mechanical ventilation: 4 (20%) received solely intratracheal ventilation and 2 (10%) CPAP; 4 (20%) children received both types of ventilation.

### Influenza-associated severe complications and outcome

Thirty-nine severe influenza-associated complications and two fatalities were reported among the 20 children (Table 3). The most frequently reported complication was influenza-associated pneumonia in 12 (60%) patients, followed by bronchitis/bronchiolitis in 6 patients (30%). Secondary bacterial pneumonia was observed in 5 (25%) patients; in 4 cases in combination with influenza-associated pneumonia. ARDS was reported in 5 (25%) patients, all suffering from influenza-associated pneumonia, and in three patients together with sepsis. All 5 patients with ARDS had predisposing underlying chronic conditions. Encephalitis/encephalopathy occurred in a total of 5 (25%) patients (including one fatal case), in three cases in combination with bronchitis/bronchiolitis.

**Table 3:** Severe influenza-associated complications and fatalities in children treated in PICUs - Germany, 2005-2008 *

Severe complications/death	No. of children (%)
Influenza-associated pneumonia	12 (60)
Bronchitis / bronchiolitis	6 (30)
Secondary bacterial pneumonia	5 (25)
Encephalitis / encephalopathy	5 (25)
ARDS	5 (25)
Sepsis	3 (15)
Status asthmaticus	2 (10)
Apnoea / bradycardia	1 (5)
Death	2 (10)

* Data on diagnoses were available from 20 children; percentages refer to the number of the available patients. Multiple nominations were possible.

Of the two (10%) reported fatalities, one was an 8-year-old boy admitted to the PICU with suspected encephalitis and status epilepticus in 2007. Previously he was diagnosed with a delay in speech development and suspected rolandic epilepsy. He received antibiotics, catecholamines, acyclovir and intratracheal ventilation. He was diagnosed with influenza A one day after PICU admission and was started on oseltamivir treatment. He died 4 days after admission from massive cerebral edema. The autopsy report stated death due to cardiac and circulatory failure. *Streptococcus pneumoniae *was diagnosed from PCR analysis of cerebrospinal fluid and brain tissue, indicating encephalopathy following influenza infection complicated by bacterial superinfection of the CNS. In 2008, a second fatality was reported from a 13-year-old boy with several chronic underlying diseases (asthma bronchiale, cardiac malformation, Addison's disease). He was found unconscious by his parents after febrile infection during the previous week. After resuscitation he was transported to the hospital where he died in the emergency room from cardiac and respiratory failure. The autopsy report diagnosed cerebral edema and PCR-confirmed seasonal influenza A (H1N1) from lung biopsy tissue.

Permanent or possibly permanent sequelae were described in 2 (10%) and 7 (35%) patients, respectively. Permanent sequelae were worsening of an underlying chronic lung disease (BPD) in a patient after influenza A-associated pneumonia, and post-encephalitic/organic brain syndrome in a patient with influenza A-associated encephalopathy. Possible permanent sequelae occurred in 5 patients after influenza A and in 2 patients after influenza B. Reported possible sequelae were pulmonary callosity following secondary bacterial pneumonia complicated by pleural effusion (2 patients), neurological sequelae (ataxia; severe illness polyneuropathy) following encephalopathy (2 patients), regression in locomotion development following PICU treatment for almost 2 months (1 patient), and postural damage after pleural decortication (1 patient); in one patient the sequelae were not specified.

### Underlying chronic conditions and risk factors

Underlying chronic medical conditions were reported in 11 (55%) children, whereby 7 of these had more than one such condition. Chronic pulmonary diseases were most frequent and were reported for 8 (40%) children; three had asthma bronchiale but no other pulmonary disease, three had possible broncho-pulmonary dysplasia (BPD) and two had other pulmonary diseases. Further underlying medical conditions were: cardiac malformations in 4 (20%), immunocompromised condition in 4 (20%), preterm birth in 2 (10%), and other chronic conditions in 4 (20%) children (Addison's disease; Cornelia-De-Lange Syndrome; hereditary motor and sensory neuropathy (HMSN) and scoliosis; unclear retardation). Patients with underlying chronic conditions stayed significantly longer in the hospital (median 31 days, IQR 18-55) than patients without underlying conditions (median 13 days, IQR 7-19; p = 0.01). They also tended to stay longer at the PICU (p = 0.06), and a higher proportion of these children needed mechanical ventilation (73% vs. 22%, p = 0.07).

Information about the influenza vaccination status was available from 17 (85%) patients; none had been vaccinated against influenza. Out of the 11 children from risk groups with chronic underlying disease the vaccination status was not available in 2 (18%) patients, and 1 (9%) child was < 6 months of age and therefore too young to be immunized.

## Discussion

We describe the results from a nation-wide surveillance study on severe paediatric seasonal influenza cases in Germany during three pre-pandemic influenza seasons between 2005 and 2008. A total of 20 laboratory-confirmed influenza-associated PICU cases (14 influenza type A, 5 type B, 1 type unknown), including two fatalities, were reported. This number was surprisingly low and corresponded to an estimated annual incidence for PICU-admitted children with seasonal influenza of 0.1/100,000 children < 5 years of age. In contrast, for the same age group incidences of PICU admissions associated with seasonal influenza were 4/100,000 in USA and 1-13/100,000 in South Australia [5,7]. Apart from country-specific differences in access to health care as well as in criteria for ICU hospitalisation, the lower incidence found in our study may also be due to the weak to moderate influenza activity during the observation period in Germany [11]. Another important explanation is possible under-diagnosis of influenza in the hospitals, as not all physicians perform influenza testing of children admitted with severe acute respiratory infection. Low participation in the voluntary reporting system due to frequent changes of the medical staff in the PICUs is a further possible reason. Hence, the low reported incidence for PICU-admitted influenza cases in this study may underestimate the true incidence of severe paediatric influenza cases in Germany. With regard to fatal paediatric cases it should be noted that our study was not designed to capture influenza-associated fatalities that occurred outside PICUs, e.g. at home or during emergency treatment. For comparison, the national mortality register reported a total of 25 influenza-associated (ICD-10 code J10) deaths in children < 15 years of age during the years 2006 to 2008.

Our study showed that in Germany children < 1 year of age and children with chronic underlying diseases are the main paediatric risk groups for severe seasonal influenza infections. Influenza-associated pneumonia, secondary bacterial infections, ARDS and encephalitis/encephalopathy were frequent complications. The observed clinical characteristics were similar to the results from the U.S. and other countries [2,4,8,12-17]. However, children in our study were older (median 7.5 years) than in an ICU study in the U.S. (median 1.5 years) [12]. Fatalities also were reported only in two older children (8 and 14 years of age), whereas the majority of reported paediatric fatalities in the U.S. and Australia are below 5 years of age [14,17]. In our study, ARDS occurred in 25% of the PICU children, in contrast to a retrospective chart review in an U.S. children's hospital with 27 PICU children diagnosed with seasonal influenza 2008/2009 [18], which reported only 4% of ARDS.

In the current study, 10 (50%) patients were treated with oseltamivir, whereby in 5 (25%) patients treatment was started several days after the appearance of influenza symptoms or ICU admission. Antiviral treatment is most beneficial when started within 2 days after onset of symptoms. Nevertheless, oseltamivir treatment initiated later than 2 days after the first influenza symptoms is still considered useful in children with influenza pneumonia or to reduce viral shedding in the hospital [19]; it also seems to reduce the risk of secondary bacterial super-infections, although the mechanisms are not fully understood [13]. In our study, co-infection with more than one bacterial species was found only in patients that did not receive oseltamivir treatment.

In total, 35% of the patients suffered from bacterial co-infection, including one fatality associated with *Streptococcus pneumoniae*. Patients with bacterial or fungal co-infections stayed twice as long (median 18 days) at the PICU than other influenza patients, thus indicating the high influence of those co-infections on the morbidity. *Staphylococcus aureus *was the most frequently observed bacterial species. The data from Germany indicate a similar situation as observed on severe paediatric influenza cases (fatalities) in the USA, which showed bacterial co-infections in 34% of patients in 2007, most frequently with *Staphylococcus aureus *[13].

In Germany, influenza vaccination in children is currently recommended for risk groups, defined by chronic underlying diseases. In 2005, a regional survey in children < 24 months of age showed that 5% of all children had been vaccinated against influenza [20]. Among the severe cases reported in our study no child had been vaccinated. Eleven (55%) belonged to risk groups with chronic underlying disease, including eight (40%) of at least two years of age for whom influenza vaccines are considered to be efficacious [21]. One risk group child was below 6 months of age and, hence, too young to be immunized. Two other risk group children were under two years of age; for this age group, the efficacy of the inactivated influenza vaccines licensed in Germany is unclear, and data on safety are insufficient thus far [21]. Forty-five% of the patients with severe influenza were previously healthy children, who would not be covered by a risk-based vaccination strategy. A recommendation to vaccinate healthy children routinely against seasonal influenza is not widespread in Europe, but has been argued to have potential community benefits [22].

Renewed interest in paediatric influenza arose with the occurrence of the new pandemic influenza A (H1N1) 2009 in Germany from April 2009 onwards [23]. An ongoing ESPED study on children with severe pandemic influenza A (H1N1) 2009 reported 93 cases below 15 years of age from 55 PICUs during a single season (August 2009 to April 2010), including 15 fatalities (11 within PICUs) [24]. This high number of cases reported in the ongoing ESPED study may be partly due to an increased awareness in physicians regarding the new subtype of influenza with unclear severity at the start of the pandemic, resulting in more frequent laboratory testing and diagnosis of influenza [5,24]. Additionally, the higher number of PICU admissions may be due to a truly higher severity of the pandemic influenza A (H1N1) 2009 subtype in children compared to previous seasonal influenza subtypes [e.g., [18,25]], although not all studies report such a difference in severity [26,27], or found a difference only regarding risk groups [28]. Thus, a comparison of both ESPED studies on the absolute number of severe influenza cases [24] has to be regarded with caution, and may underestimate the importance of severe seasonal influenza caused by non-pandemic influenza virus subtypes in children in Germany. Further prospective, laboratory-confirmed surveillance in PICUs is needed to clarify the complication rates associated with influenza virus A and B and their different subtypes and lineages.

## Conclusions

Almost half of the children with severe complications associated with seasonal influenza were previously healthy children, for whom there is currently no recommendation to vaccinate against influenza. Fifty% were children with chronic diseases for whom vaccination had been recommended but had not been followed. In these children the severe course of disease might have been prevented by vaccination. Oseltamivir treatment was often started several days after start of influenza symptoms or PICU admission, indicating that influenza is often diagnosed too late for effective antiviral patient treatment. The general incidence of influenza-associated severe disease in German PICUs was very low, but under-diagnosis and under-reporting during these pre-pandemic influenza seasons may be likely. Therefore prospective studies providing influenza subtype screening on PICU admissions of children with respiratory disease are needed to assess more accurately the burden of severe influenza-associated complications. These studies are necessary to provide data and evidence for the discussion on influenza prevention by general routine vaccination in children.

## List of abbreviations used

ARDS: acute respiratory distress syndrome; BPD: broncho-pulmonary dysplasia; CNS: central nervous system; CPAP: continuous positive airway pressure; CRP: C-reactive protein; ESPED: "Erhebungseinheit Seltener Pädiatrischer Erkrankungen in Deutschland" [Surveillance Unit of Rare Paediatric Diseases in Germany]; HMSN: hereditary motor and sensory neuropathy; ICD-10: International Classification of Diseases, 10^th ^revision; ICU(s): intensive care unit(s); IQR: inter-quartile range; PCR: polymerase chain reaction; PICU(s): paediatric intensive care unit(s); PRIDE: "Pädiatrische Respiratorische Infektionen in Deutschland" [Paediatric; Respiratory Infections in Germany]; RSV: respiratory syncytial virus; sp.: species; SPSS: Statistical Package for the Social Sciences; U.S.: United States; vs.: versus

## Competing interests

All authors have received grants for studies, lecturing fees or travelling grants from the study sponsor Novartis Vaccines and Diagnostics GmbH & Co. KG, or from other vaccine manufactures (GlaxoSmithKline (AS, VG, JGL), Sanofi Pasteur MSD (AS, JGL), AstraZeneca (JGL), Pfizer (JGL), and CSL Behring (JGL)) during the previous years.

## Authors' contributions

AS participated in data collection, performed the analysis, interpreted the data, and drafted the manuscript. VG designed the study, participated in data collection, and revised the manuscript. JGL supervised the study, supported data interpretation, and revised the manuscript. All authors read and approved the final manuscript.

## Pre-publication history

The pre-publication history for this paper can be accessed here:

http://www.biomedcentral.com/1471-2334/11/233/prepub
